# Maternal microchimerism accelerates immune tolerance induction to factor VIII in children with hemophilia A and FVIII inhibitors

**DOI:** 10.3389/fimmu.2026.1921432

**Published:** 2026-08-25

**Authors:** Zekun Li, Yeling Lu, Zhenping Chen, Jing Dai, Xi Wu, Xiaohong Cai, Xiaorong Pan, Siyu Cai, Gang Li, Xiaoling Cheng, Di Ai, Jialu Zhang, Qiulan Ding, Wenman Wu, Xuefeng Wang, Runhui Wu

**Affiliations:** 1 Hemophilia Comprehensive Care Center, Haematology Department, Beijing Children’s Hospital, Capital Medical University, National Center for Children’s Health, Beijing, China; 2 Clinical Laboratory Medicine Department, Ruijin Hospital, Shanghai Jiaotong University School of Medicine, Shanghai, China; 3 Cell and Gene Therapy Laboratory, Beijing Pediatric Research Institute, Beijing Children’s Hospital, Capital Medical University, National Center for Children’s Health, Beijing, China; 4 Blood Transfusion Department, Ruijin Hospital, Shanghai Jiaotong University School of Medicine, Shanghai, China; 5 Faculty of Laboratory Medicine, Ruijin Hospital, Shanghai Jiaotong University School of Medicine, Shanghai, China; 6 Center for Clinical Epidemiology and Evidence-based Medicine, Capital Medical University, Beijing, China; 7 Department of Pharmacy, Beijing Children’s Hospital, Capital Medical University, National Center for Children’s Health, Beijing, China; 8 Pediatrics Departments, National Key Discipline of Pediatrics, Capital Medical University, Beijing, China; 9 Collaborative Innovation Center of Hematology, Shanghai Jiaotong University School of Medicine, Shanghai, China

**Keywords:** FVIII, hemophilia A, immune tolerance induction, inhibitor, maternal microchimerism

## Abstract

**Background:**

Predictors for the successful eradication of neutralizing anti-FVIII alloantibodies (inhibitors) in hemophilia A patients receiving immune tolerance induction (ITI) therapy remain limited. Maternal microchimerism (MMc) showed potential to protect hemophilia A patients from inhibitor development.

**Objectives:**

To investigated the role of MMc in ITI therapy.

**Patients/Methods:**

This observational study enrolled 121 pediatric with hemophilia A and inhibitors. MMc was determined using droplet digital PCR. Low-dose ITI (FVIII ~50IU/kg every other day) was administered, with adjunctive rituximab given to patients with higher risk clinical features.

**Results:**

Of the 101 patients evaluable for MMc, 88 completed ITI, 18 were MMc positive (MMc+) and 70 were MMc negative (MMc-). Success was achieved in 75 (85.2%) patients, including 16 of 18 MMc+ patients (88.9%) and 59 of 70 MMc- patients (84.3%). Compared with MMc- patients, MMc+ patients had a lower peak inhibitor during ITI (median, 5.3 vs. 37.8 BU/ml, *p* = 0.029), less frequent rituximab use (27.8% vs. 61.4%; *p* = 0.011), and a shorter time to ITI success (median, 3.0 vs 9.9 months; *p* = 0.009). Multivariate Cox regression identified MMc+ (HR = 2.770), pre-ITI inhibitor <10BU/ml (HR = 2.663), peak inhibitor during ITI<200BU/ml (HR = 4.954) and non-large deletion/duplication *F8* mutations (HR = 2.344) as independent predictors of shorter time to ITI success.

**Conclusion:**

MMc was associated with more rapid ITI success in children with hemophilia A and inhibitors receiving low-dose ITI regimen, suggesting the potential role of MMc in facilitating the eradication of FVIII inhibitors.

## 1 Introduction

The development of factor VIII (FVIII) neutralizing antibody (inhibitor) remains a major complication in hemophilia A (HA) treatment, as it renders FVIII replacement therapy ineffective (1). Inhibitor is associated with increased bleeding episodes, more chronic pain and worse musculoskeletal damage (2). Although the need for inhibitor eradication is debated in the era of non-factor prophylaxis, it remains a desirable goal to enable effective FVIII-based hemostasis during surgery or acute bleeding and to preserve potential eligibility for future gene therapy (3, 4).

Immune tolerance induction (ITI), involving prolonged exposure to high dose FVIII is the standard therapy of inhibitor eradication (5), with reported success rates of 60-90% (5). Data from a US cohort of 485 patients showed that ITI was successful in almost 60% of cases, whereas partial success and failure each occurred in approximately 20% (2). Genetic, patient and inhibitor-related factors may influence ITI outcomes. For example, large deletions in *F*8 *(*6) and higher plasma level of anti-FVIII IgG4 (7) have been associated with poorer ITI outcomes. Furthermore, meta-analysis showed that lower historical peak inhibitor, pre-ITI inhibitor and peak inhibitor during ITI were associated with a higher probability of ITI success (8).

Genetic factors related with ITI outcomes remain incompletely understood, although they may enable outcome prediction before ITI initiation. Identified genetic factors and biomarkers associated with primary tolerance (inhibitor development) included HLA allele subtype, single nucleotide polymorphism in immune response genes such as IL-10, TNFA, CTLA-4 (9) and BAFF (10), as well as the expression levels of innate immune modulators (11, 12). The reported factors related with secondary tolerance (inhibitor eradication) include BAFF expression level (13), humoral immune response, and factors involved in complement and coagulation cascades (14).

We previously reported that maternal microchimerism (MMc), detected in peripheral blood cells using sensitive droplet digital polymerase chain reaction (ddPCR), was associated with a significantly lower risk of inhibitor development (15). Trace amounts of FVIII expressed by MMc cells were proposed to be non-inherited maternal antigen and to promote immune tolerance toward FVIII in HA patients. However, some patients still developed inhibitors despite the presence of MMc. It is unclear how MMc impacts the severity of inhibitor complications and response to the ITI therapy. Therefore, in this study, we investigated MMc status in pediatric HA patients with inhibitors and evaluated the role of MMc in ITI outcomes.

## 2 Materials and methods

### 2.1 Study design and participants

This observational study was approved by the ethics committee of Beijing Children’s hospital, Capital Medical University. Written informed consent was obtained from all guardian of patients. Boys with hemophilia A, positive of FVIII inhibitor: ≥0.6BU/ml in two continuous testing, no allergic reaction to FVIII concentrates, agree to test *F8* mutation were sought from January 2017 to July 2021.

### 2.2 ITI therapy

All patients received low-dose ITI with plasma derived FVIII (FVIII: von Willebrand factor ratio of 1:0.7) at 50 FVIII IU/kg every other day. Patients were treated either with ITI-alone, or ITI combined with rituximab (ITI-Ritux) if they were unresponsive or expected with poor response to ITI-alone therapy (**Supplementary Methods**).

The ITI-Ritux protocol included rituximab (375 mg/m^2^, max 600 mg, weekly for 4 weeks) and adjunctive prednisone (2 mg/kg, max 60 mg, daily for 1 month, tapered over 3 months). For patients showing poor-outcome event (s) after the 1^st^ round rituximab, additional rounds were administered at the discretion of investigator. (**Supplementary Methods**). Treatment decisions were made without knowledge of MMc status.

### 2.3 Outcome, definition and laboratory measurements

Successful ITI was defined as (1) negative inhibitor titer (<0.6 BU/ml) on two consecutive measurements; (2) normal FVIII recovery (≥66% of expected), and (3) sustained negative inhibitor titer for ≥6 months (ITI-alone regimen) or ≥12 months from the last round rituximab (ITI-Ritux regimen) (14). For patients achieving success, time to ITI success was defined as the duration from ITI initiation to the first negative inhibitor titer. Failure was defined as not achieving success after at least 24 months of ITI or by clinical assessment of early failure.

### 2.4 Maternal microchimerism determination

The presence of MMc was assessed using ddPCR, adapted from our previously described method (15). Briefly, the genomic DNA was extracted from peripheral blood leukocytes using the QIAamp DNA Blood kit (Qiagen, Hilden, Germany), and DNA concentrations were measured using NanoDrop 2000 spectrophotometer (Thermo Fisher Scientific, Wilmington, DE, USA). DNA samples were diluted to 50ng/μl and digested with the KpnI restriction enzyme (Takara, Japan).

Three X chromosome single nucleotide polymorphism (SNPs) with high minor allele frequencies in the Chinese population were selected as markers. Commercially available predesigned TaqMan SNP Genotyping Assays (Thermo Fisher Scientific) were used: rs2885051 (C/G, Assay ID: C:26367236_20), rs5961905 (C/T, C:30604214_10), and rs5910801 (A/C, C:30481314_20). Heterozygosity at these three loci was screened via qPCR using TaqMan Master Mix (Invitrogen, Thermo Fisher Scientific, USA). Patient-mother pairs were considered informative for MMc analysis if the mother was heterozygous for a maternal-specific allele at any of the three loci. Pairs in which the mother was homozygous for all three SNPs were excluded from the analysis.

ddPCR was performed using the QX200 system (Bio-Rad Laboratories, Hercules, CA, USA) and the same TaqMan SNP Genotyping Assays to detect maternal-specific alleles in genomic DNA from patients’ peripheral blood leukocytes. Approximately 180 ng DNA, corresponding to ~27, 000 X-chromosomal target copies in male leukocyte, was loaded per reaction, consistent with the manufacturer’s guidance for rare-allele detection (16). Patients were considered MMc-positive if maternal-specific alleles were detected.

Thermal cycling conditions were: activation at 95 °C for 10 min (1 cycle); denaturation at 94 °C for 30 s followed by annealing at either 60 °C (for rs2885051) or 58 °C (for rs5910801 and rs5961905) for 1 min (40 cycles); and enzyme deactivation at 98 °C for 10 min (1 cycle), followed by a hold at 4 °C. Data were quantitatively analyzed using QuantaSoft software. Results are presented as the ratio of droplets positive for the maternal-specific SNP to droplets positive for the shared SNP.

### 2.5 Statistical analysis

Statistical analyses were performed using SPSS version 22.0 (IBM Corp., Armonk, NY, USA) and GraphPad Prism version 9.0 (GraphPad Software, La Jolla, CA, USA). Continuous variables were compared using the Student’s t-test for normally distributed data or the Mann-Whitney U test for non-normally distributed data. Categorical variables were presented as frequencies and percentages and compared using the Pearson’s chi-square test or Fisher’s exact test. Among MMc-positive patients, associations of MMc level with peak inhibitor during ITI and age at ITI initiation were assessed using Spearman’s rank correlation, and its association with time to ITI success using univariable Cox regression.

Kaplan–Meier curves with log-rank tests were used to evaluate prognostic factors for time to ITI success; significant variables were subsequently entered into a multivariable Cox regression model. Hazard ratios (HRs) and 95% confidence intervals (CIs) were reported. Sensitivity analyses were performed separately after excluding patients with intron 22 inversion and those with large deletion/duplication, respectively. All tests were two-sided, with P < 0.05 considered statistically significant.

## 3 Results

### 3.1 Patients enrollment and MMc detection

**Figure 1:**
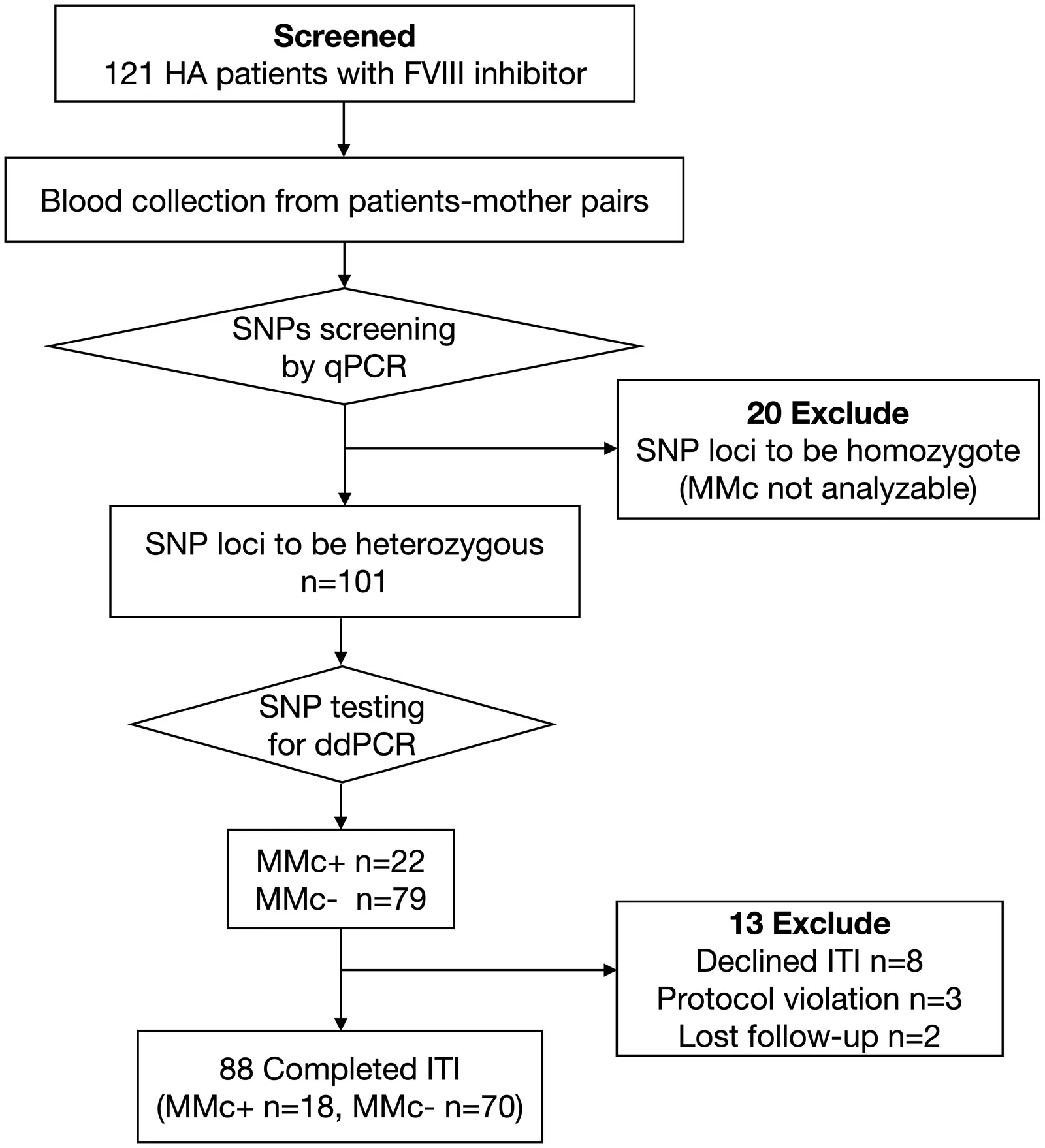
Participant flow through the study. HA, hemophilia A; ITI, immune tolerance induction; MMc, maternal microchimerism; SNP, single-nucleotide polymorphism; qPCR, quantitative PCR; ddPCR, droplet digital PCR.

A total of 121 patients with HA and inhibitors (120 with severe HA and 1 with mild HA, Supplementary Methods), along with their mothers, were sequentially screened for MMc. MMc analysis was feasible in 101 patient-mother pairs (83.5%) in which the mother was heterozygous for at least one designated SNP locus. MMc was detected in 22 of these 101 patients (21.8%), with a median chimerism level of 2.55 per 10, 000 cells (range: 1.08–24.72 per 10, 000). Of the 101 evaluable patients, 13 were subsequently excluded: 8 declined ITI, 3 had protocol violations, and 2 were lost to follow-up (**Figure 1**). The characteristics of these 13 excluded patients are detailed in **Supplementary Table 1**.

### 3.2 Demographic and baseline characteristics

A total of 88 patients with severe HA completed the study, comprising 18 MMc-positive (MMc+) and 70 MMc-negative (MMc-) patients (**Figure 1**). Genetic analysis revealed that 32 patients (36.3%) had intron 22 inversions, 3 (3.4%) had intron 1 inversions, 22 (25.0%) had nonsense mutations, 9 (10.2%) had small insertion/deletion, 14 (15.9%) had large deletion or duplication, 4 (4.5%) had splice site mutation (in conserved site), and 4 (4.5%) had unknown mutation (**Table 1**). Baseline characteristics were comparable between MMc+ and MMc- patients (**Table 1**). The cohort included four related patients from two families. In one family, both patients were MMc-; in the second family, one patient was MMc- while the other was MMc+.

**Table 1:** Demographics and baseline characteristics in 88 maternal microchimerism (MMc) analyzable hemophilia A patients with inhibitor and completed immune tolerance induction (ITI).

Variable	All patients	MMc+	MMc-	*p*-value
No. of patients, n (%)	88	18 (20.5)	70 (79.5)	–
*F8* mutation, n (%)				
- Intron 22 inversion	32 (36.4)	9 (50.0)	23 (32.9)	
- Intron 1 inversion	3 (3.4)	0 (0)	3 (4.3)	
- Nonsense mutation	22 (25.0)	5 (27.8)	17 (24.3)	
- Small insertion/deletion	9 (10.2)	1 (5.6)	8 (11.4)	
- Large deletion/duplication	14 (15.9)	1 (5.6)	13 (18.6)	
- Splice site (in conserved site)	4 (4.5)	1 (5.6)	3 (4.3)	
- Unknown mutation	4 (4.5)	1 (5.6)	3 (4.3)	
Age at inhibitor detection, yr	2.6 (0.7, 12.7)	1.8 (0.8, 12.7)	2.7 (0.7, 10.1)	0.275
Exposure days at inhibitor detection	30 (4, 200)	32.5 (7, 109)	29 (4, 200)	0.597
Age at ITI initiation, yr	3.7 (0.5, 12.7)	2.7 (0.9, 12.7)	4.1 (0.5, 12.0)	0.108
Time interval from inhibitor diagnosis to ITI, mo	5.0 (0, 112.6)	2.8 (0.2, 34.7)	6.1 (0, 112.6)	0.508

Continuous variables are presented as median (range). yr, years; mo, months.

### 3.3 ITI therapy and outcomes

Among the 88 patients completed ITI therapy, the median age at ITI initiation was 3.7 years (range 0.5-12.7), the interval from inhibitor detection to ITI initiation was median 5.0 months (range, 0–112.6). Forty patients (45.5%) received ITI-alone throughout treatment, whereas 48 (54.5%) received ITI-Ritux. In the ITI-Ritux group, 30 patients received rituximab upfront (MMc+, n = 5; MMc−, n = 25), 18 patients, all MMc−, initially received ITI-alone and subsequently received rituximab because of an inadequate inhibitor response. One, two, three, and four rounds of rituximab were administered to 35 (72.9%), 9 (18.8%), 3 (6.3%), and 1 (2.1%) patients, respectively. The median follow-up duration was 29.7 months (IQR, 15.7–44.3), and 75 patients (85.2%) achieved ITI success.

### 3.4 ITI efficacy according to MMc status

**Figure 2:**
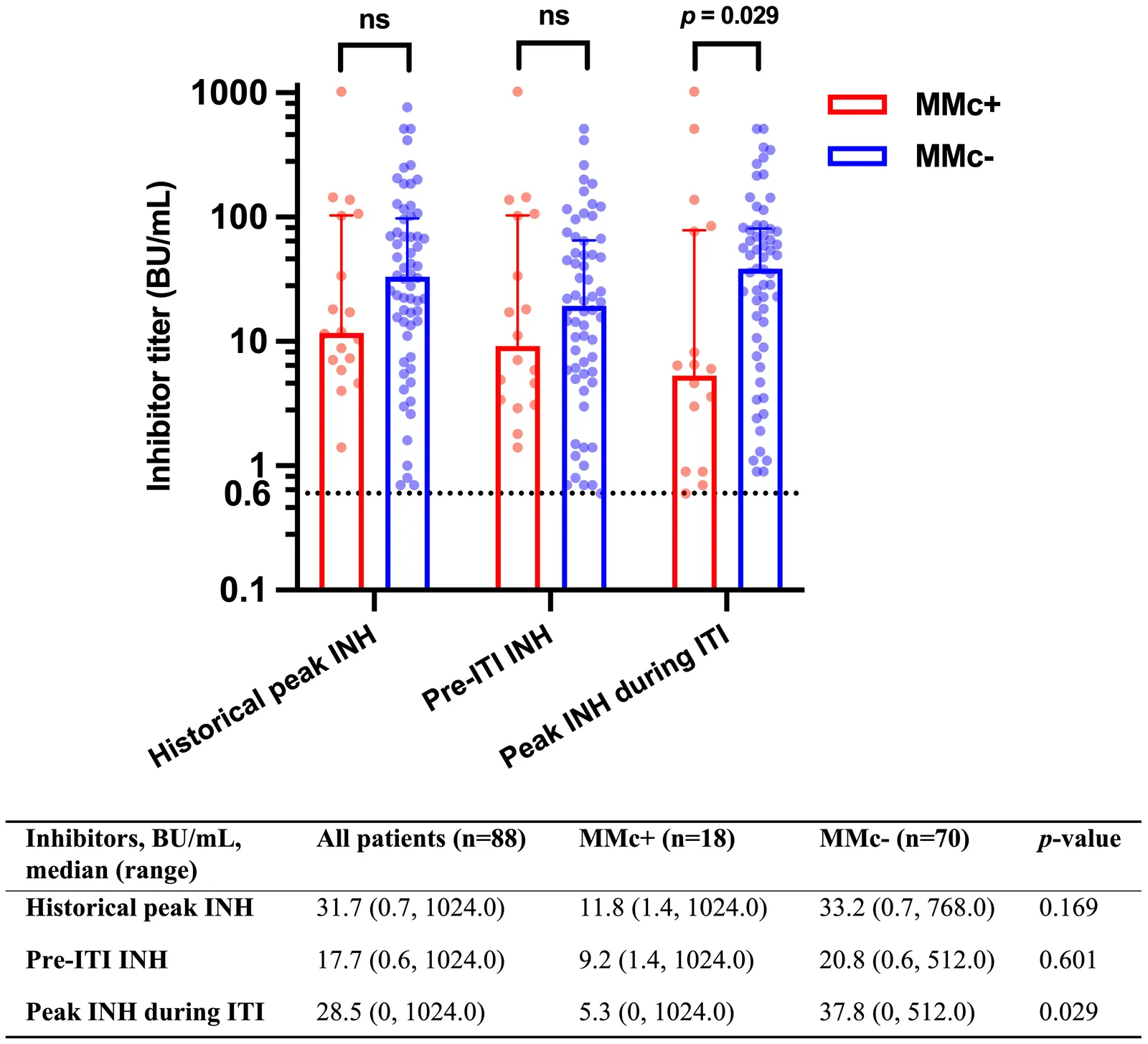
FVIII inhibitor titers according to MMc status. Patients were stratified according to MMc status into the MMc-positive group (MMc+, n = 18) and the MMc-negative group (MMc−, n = 70). Historical peak inhibitor, pre-ITI inhibitor, and peak inhibitor during ITI are presented on a logarithmic scale. Group comparisons were performed using the two-sided Mann–Whitney U test. Compared with MMc− patients, MMc+ patients had a significantly lower peak inhibitor titer during ITI (*p* = 0.029), whereas no significant differences were observed in historical peak inhibitor or pre-ITI inhibitor. Each point represents an individual patient. The bars and error bars represent the median and interquartile range. The horizontal dotted line indicates the inhibitor-negativity threshold of 0.6 BU/mL. MMc, maternal microchimerism; INH, inhibitor; ITI, immune tolerance induction; BU, Bethesda units.

**Figure 3:**
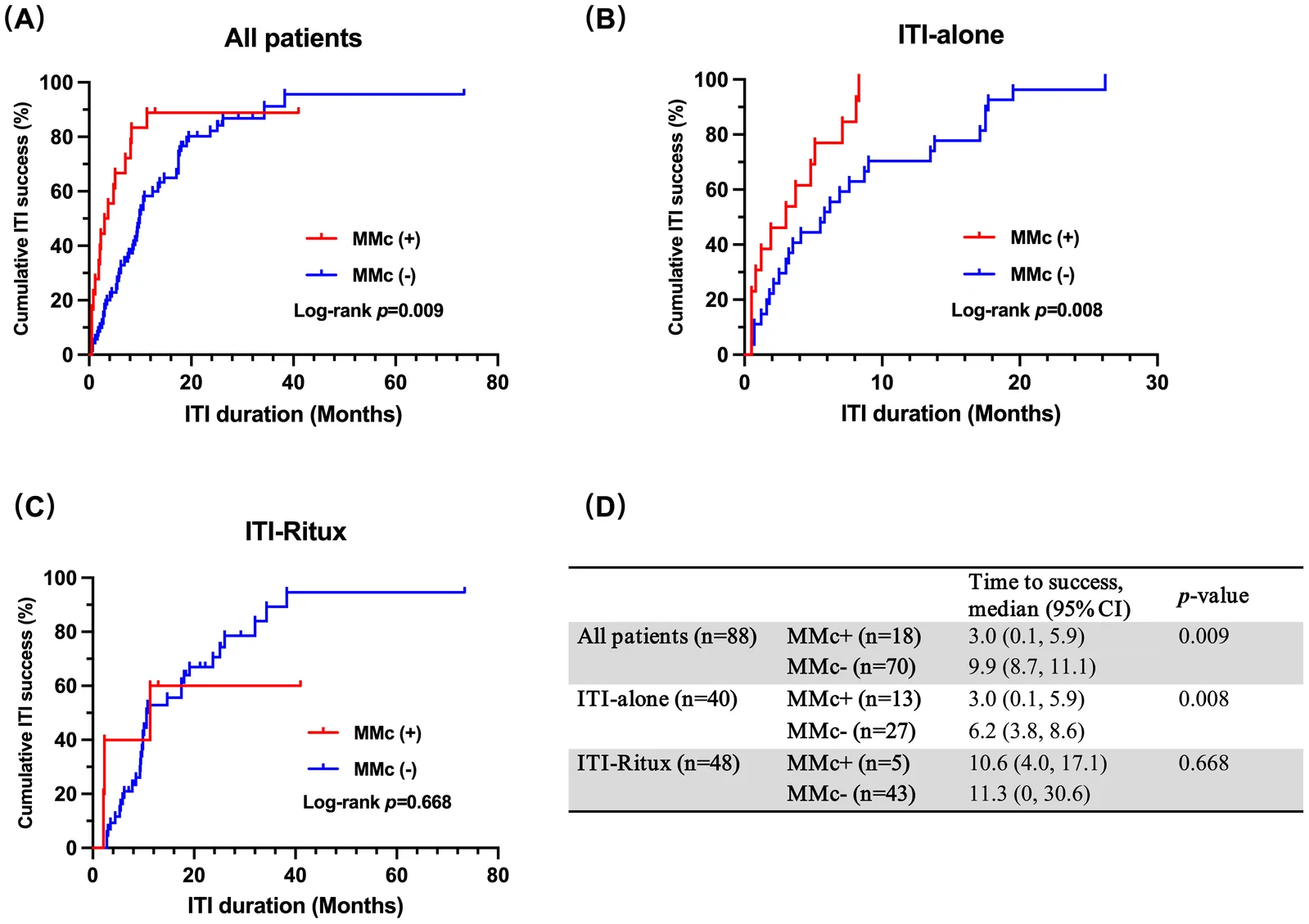
Cumulative ITI success according to MMc status. Kaplan–Meier curves with log-rank tests were performed for **(A)** all patients (n = 88; MMc+, n = 18; MMc−, n = 70), **(B)** patients receiving ITI-alone (n = 40; MMc+, n = 13; MMc−, n = 27), and **(C)** patients receiving ITI combined with rituximab (ITI-Ritux; n = 48; MMc+, n = 5; MMc−, n = 43). MMc+ patients had a significantly shorter time to ITI success than MMc− patients in the overall cohort (*p* = 0.009) and the ITI-alone group (*p* = 0.008), whereas no significant difference was observed in the ITI-Ritux group (*p* = 0.668). **(D)** Summary of the estimated median time to ITI success. MMc, maternal microchimerism; ITI, immune tolerance induction; Ritux, rituximab.

ITI success rate was 88.9% (16/18) for MMc+ patients and 84.3% (59/70) for MMc- patients. The peak inhibitor during ITI was significantly lower in MMc+ than in MMc- patients (median, 5.3 BU/ml [range, 0–1024.0] vs. 37.8 BU/ml [range, 0–512.0]; *p* = 0.029). No significant differences were observed in historical peak or pre-ITI inhibitor between the groups (**Figure 2**). MMc+ patients received the ITI-Ritux regimen less frequently than MMc- patients (27.8% vs. 61.4%; *p* = 0.011). Notably, MMc+ patients had a significantly shorter time to ITI success than MMc− patients (median, 3.0 months [95% CI, 0.1–5.9] vs. 9.9 months [95% CI, 8.7–11.1]; *p* = 0.009; **Figures 3A, D**).

All 40 patients who received ITI-alone achieved success. In this group, MMc+ patients had a significantly shorter time to ITI success than MMc− patients (median, 3.0 months [95% CI 0.1–5.9] vs. 6.2 months [95% CI 3.8–8.6]; *p* = 0.008; **Figures 3B, D**). Among the 48 patients in the ITI-Ritux group, 35 (72.9%) achieved ITI success. Neither the success rate (60.0% [3/5] vs. 74.4% [32/43]; *p* = 0.602) nor the estimated median time to ITI success (10.6 vs. 11.3 months; *p* = 0.668) differed significantly between the MMc+ and MMc- patients (**Figures 3C, D**).

Among the 18 MMc+ patients, MMc levels were not significantly associated with peak inhibitor during ITI (Spearman’s ρ = 0.282, *p* = 0.257), time to ITI success (HR = 0.980, 95% CI, 0.894–1.075; *p* = 0.675), or age at ITI initiation (Spearman’s ρ = 0.160, *p* = 0.525).

### 3.5 Predictors of ITI outcomes

Univariate analysis using Kaplan-Meier curves and log-rank tests showed that MMc+ (*p* = 0.009), historical peak inhibitor <200 BU/mL (*p* = 0.013), pre-ITI inhibitor <10 BU/mL (*p* < 0.001), peak inhibitor during ITI <200 BU/mL (*p* = 0.001), and non-large deletion/duplication *F8* mutations (*p* = 0.001) were associated with ITI success. When analyzed as a continuous variable, age at ITI initiation was not associated with time to ITI success in univariable Cox regression (*p* = 0.842).

In the multivariable Cox regression analysis, MMc+ (hazard ratio [HR] =2.770; *p* = 0.001), pre-ITI inhibitor <10BU/ml (HR = 2.663; *p* < 0.001), peak inhibitor during ITI <200BU/ml (HR = 4.954; *p* = 0.005) and non-large deletion/duplication *F8* mutations (HR = 2.344; *p* = 0.039) were identified as significant independent predictors of ITI success (**Table 2**).

**Table 2:** Univariate and Multivariate regression model of ITI outcome.

Univariate analysis				*p-*value
*F8* mutation (large deletion/duplication and others)				0.001
MMc (+/-)				0.009
Historical peak inhibitor (<200BU/ml vs ≥200BU/ml)				0.013
Age at ITI start (<5 years vs ≥5 years)				0.383
Time interval from inhibitor detection to ITI (<12 months vs ≥12 months)				0.755
Pre-ITI inhibitor (<10BU/ml vs ≥10BU/ml)				<0.001
Peak inhibitor during ITI (<200BU/ml vs ≥200BU/ml)				0.001

Multivariate analysis		HR (95% CI)			*p*-value
MMc+	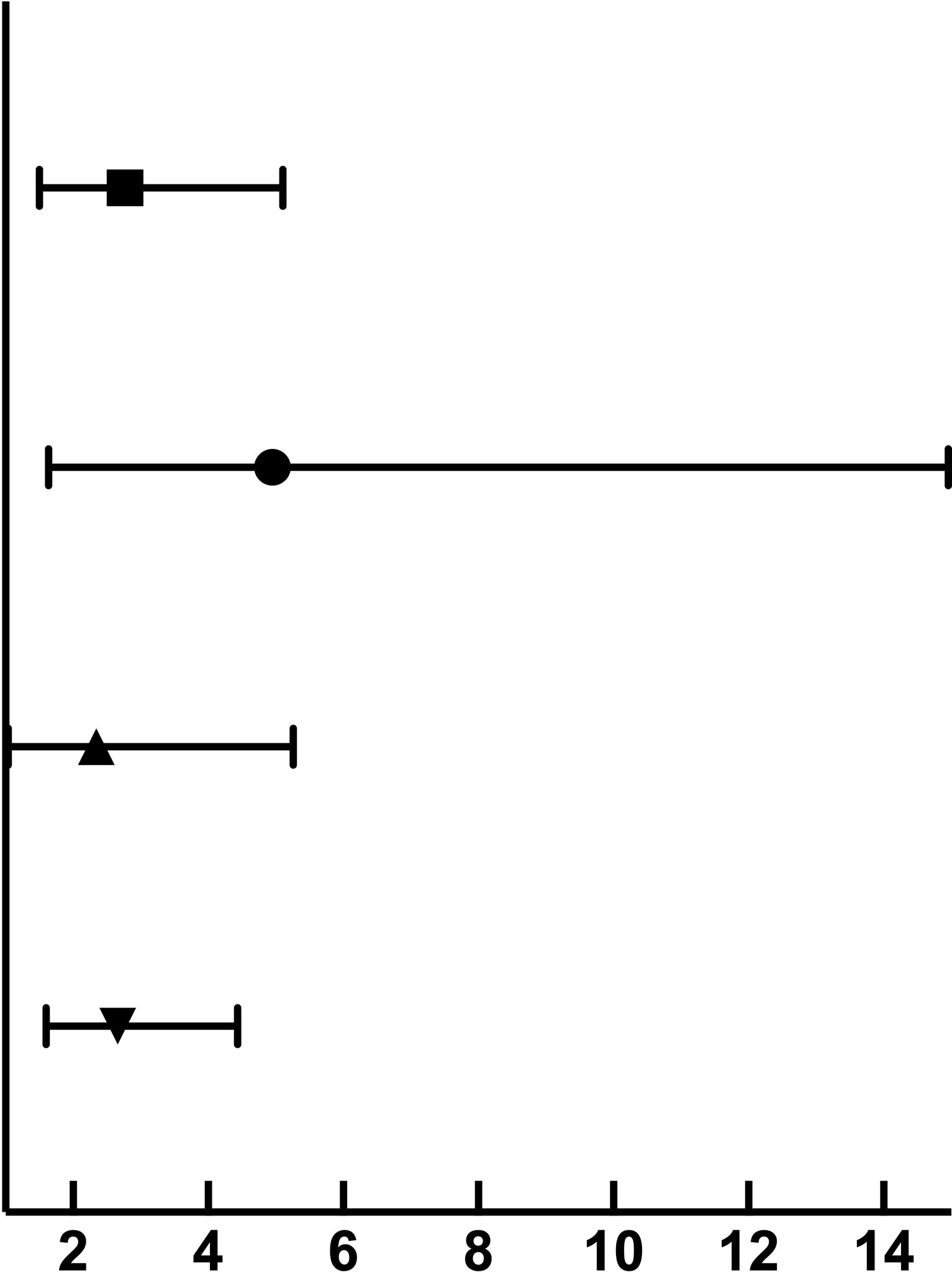		2.770 (1.501, 5.111)	0.001
Peak inhibitor during ITI <200BU/ml			4.954 (1.640, 14.961)	0.005
Non-large deletion/duplication *F8* mutations			2.344 (1.044, 5.263)	0.039
Pre-ITI inhibitor <10BU/ml			2.663 (1.599, 4.437)	<0.001

ITI, immune tolerance induction; BU, Bethesda Unit; MMc, maternal microchimerism; SE, Standard error; HR, hazard ratio. Univariate regression model was analysis by K-M survival curve.

In a sensitivity analysis excluding 32 patients with intron 22 inversion, MMc did not show a significant effect in the univariable analysis; however, it was independently associated with a shorter time to ITI success after multivariable adjustment. (**Supplementary Table 2**). In a separate analysis excluding 14 patients with large deletions/duplications, this finding remained significant in both univariable and multivariable analyses (**Supplementary Table 3**).

## 4 Discussion

This study investigated the influence of MMc on ITI outcomes in children with hemophilia A and FVIII inhibitors. Compared with MMc− patients, MMc+ patients had significantly lower peak inhibitor during ITI and achieved FVIII immune tolerance more rapidly, often without the need for additional immunosuppression. Consistent with prior reports, low pre-ITI inhibitor (<10BU/mL), lower peak inhibitor during ITI (<200BU/mL) and *F8* mutation (non-large deletion/duplication) were associated with ITI success (2, 4, 8). Moreover, multivariate analysis identified MMc as an independent predictor of successful ITI.

The association between MMc+ and rapid success was observed in the ITI-alone subgroup but not in the ITI-Ritux subgroup. The latter finding remains inconclusive because only five patients were MMc+ and the subgroup comprised patients with higher-risk clinical features. Co-administration of rituximab and prednisone may have obscured this association, and their individual effects could not be distinguished.

We previously reported that MMc was associated with a reduced risk of inhibitor development in patients with HA (15). Integrating current findings, we propose that MMc may attenuate alloimmune reactions against exogenous FVIII, thereby protecting HA patients from inhibitor development. Specifically, MMc appears to alleviate the severity of the immune response to FVIII, which could promote rapid inhibitor eradication and immune tolerance induction when low-dose ITI regimens are used.

During pregnancy, maternal cells transfer to the fetus through placenta, contributing a small amount of foreign genetic material (17). MMc cells are found in human fetal tissues from the second trimester of pregnancy, with some persisting postnatally into adulthood (18). MMc cells are reported to possess a stem cell-like cell phenotype (19, 20). In mice, MMc stem cells were detected in 46% of the fetuses, including hematopoietic stem cell, while 50% of fetuses contained maternally derived terminally differentiated cells such as endothelial cells (21). Liver sinusoidal endothelial cells are the major source of FVIII, besides, endothelial cells, hematopoietic cells can also synthesize FVIII (22). Therefore, these FVIII-synthesizing cells may contain maternally derived cells, serving as a source of trace amounts of plasma or intracellular FVIII. *In utero*, exposure to maternal antigens confers specific and lasting tolerance (23), and it is confirmed that synthesis of even minute amounts of FVIII can protect against inhibitor development (24). A study has previously demonstrated that prenatal transplantation of bioengineered human placental cells designed to produce optimized FVIII leads to a sustained elevation in plasma FVIII levels after birth, without triggering an immune response (25). Accordingly, we hypothesize that FVIII expressed by maternal cells in HA fetus *in utero* may contribute to FVIII-specific fetal tolerance.

Tolerance to non-inherited maternal antigen (NIMA) is promoted by the accumulation of immune suppressive regulatory T cells (Tregs), generated either as natural Tregs (nTregs) via central tolerance (negative selection) or as induced Tregs (iTregs) via peripheral tolerance (26). It was reported that PD-L1^+^ Treg-mediated B cell apoptosis is a main mechanism of ITI (27). However, due to the escape of some autoreactive T cells from negative selection and the high immunogenicity of the FVIII protein, even healthy subjects tolerized to FVIII can response to the FVIII and develop specific antibodies (28). We hypothesize that trace amount of FVIII expressed by maternal cells induce partial immune tolerance. Only a limited abundance and diversity of FVIII-specific T clones are still retained, while a baseline population of Tregs is established. Thus, even in cases where inhibitors develop, rapid tolerization is more likely to be achieved in MMc+ patients. Notably, pregnancy can displace pre-existing MMc and reduce NIMA-specific FOXP3+ Tregs expansion in female mice (29). This pregnancy-related loss of MMc is not applicable to boys with severe HA.

Among MMc+ patients, MMc levels were not associated with ITI outcomes. This finding should be interpreted cautiously because of the small number of MMc+ patients, the limited range of MMc levels, and measurement variability at low levels. Moreover, murine studies suggest that NIMA-specific tolerance may depend on particular maternal cell populations, rather than overall MMc levels. Depletion of LysM+ or CD11c+ cells within Vav1+ maternal leukocyte abrogated NIMA-specific FOXP3+ Treg expansion and tolerance-associated phenotypes (30). We quantified total maternal DNA in peripheral blood leukocytes but could not determine cell lineages or FVIII expression. Whether analogous tolerogenic MMc subsets exist in patients with hemophilia A remains to be established.

This study has several limitations. First, the small number of MMc+ patients warrants validation in larger, multicenter cohorts. Second, our findings derive from a pediatric Chinese cohort receiving a low-dose ITI regimen with a VWF-containing plasma-derived FVIII product. Therefore, their generalizability to adults, other ethnic populations, high-dose regimens, or other FVIII products, including recombinant and B-domain-deleted FVIII, remains unknown. Product characteristics may influence FVIII immunogenicity and ITI responses. Finally, the extremely low abundance of MMc+ cells in peripheral blood precluded characterization of their lineages and FVIII expression. Future studies will develop a hemophilia A mouse model—transferring maternal hematopoietic stem cells via direct *in utero* or maternal intravenous injection—to evaluate offspring chimerism and FVIII expression. These mechanistic explorations may aid in developing novel immune tolerance strategies based on maternal-fetal cell transfer.

## 5 Conclusion

This study suggested that MMc is associated with improved responses to ITI therapy in hemophilia A patients with inhibitors. Further mechanistic studies are warranted to elucidate the underlying processes and assess the therapeutic implications of MMc.

## Data Availability

The dataset generated during the current study is available in figshare, DOI 10.6084/m9.figshare.33264711.
