# Supplementary material for: Maternal microchimerism accelerates immune tolerance induction to factor VIII in children with hemophilia A and FVIII inhibitors
Source: Front Immunol. 2026 Aug 25;17:1921432. doi: 10.3389/fimmu.2026.1921432 (PMC13549857; doi:10.3389/fimmu.2026.1921432)
Supplement: Supplementary file 1 [file SupplementaryFile1.docx]

**Supplementary** **materials**

**Title: Maternal Microchimerism Accelerates Immune Tolerance Induction to Factor VIII in Children with Hemophilia A and FVIII Inhibitors**

**Methods**

**Criteria for ITI-** **rituximab**

Rituximab and prednisone were applied in patients unresponsive or expected with poor response to ITI-alone therapy:

(a) Rituximab was added up front with ITI when (i) the patient’s historical peak inhibitor titer was ≥100 BU, or (ii) when the inhibitor tier at ITI initiation was ≥40 BU;

(b) Rituximab was added during low-dose ITI when (i) the patient’s inhibitor titer increased to ≥40 BU (ii) the patient’s inhibitor titer did not show a steady decreasing tendency (decrease in two consecutive times) after the first 3 months ITI (iii) the patient’s inhibitor titer failed to decline by >20% in any subsequent 3-month period after the initial peak.

**Criteria for additional courses of rituximab**

For patients who showed poor-outcome event(s) after the 1^st^ round rituximab, additional rounds (2^nd^ to 4^th^ rounds, 1-4 weeks for each round) were administered at the discretion of investigator

Poor-outcome events include:

(a) Insufficient inhibitor-titer decay: if one of these conditions occurred

(i) Inhibitor titer did not decline by 20% in any consecutive 6 months after starting rituximab.
(ii) Inhibitor continued to increase for more than 2 months and by more than 1.5BU above the lowest titer.
(iii) Inhibitor titer remained at >200BU by month 4 or >100BU by month 6 after rituximab administration.

(b) Relapse: When a patient who has achieved IN, the inhibitor titer increased to above 0.6 BU for 2 consecutive times.

**Classification of Hemophilia A Severity**

Among the 121 screened patients, 102 were locally classified as having severe hemophilia A, 18 as moderate, and 1 as mild. The 18 moderate patients were reclassified as severe because 17 carried *F8*-null variants and 1 had an unknown *F8* variant but a trough FVIII level <1% during prophylaxis after successful ITI. The patient with mild hemophilia A carried an F8 missense variant, c.6046C>T (p.Arg2016Trp), and remained classified as mild.

**Table 1. Characteristics of the 13 excluded patients with detectable MMc.**

| No. | MMc | chimerism level (‱) | F8 mutation | Age at inhibitor diagnosis, yr | Age at start of ITI (screen), yr | Time interval from inhibitor diagnosis to ITI (screen), mo | Historical peak inhibitor titer, BU/mL | Pre-ITI (screen) inhibitor titer, BU/mL | Peak inhibitor titer during ITI, BU/mL | Reason of withdraw | ITI regimen, outcome, time to negative inhibitor |
| --- | --- | --- | --- | --- | --- | --- | --- | --- | --- | --- | --- |
| 1 | pos | 2.54 | Intron 22 inversion | 1.0 | 1.2 | 2.0 | 17.2 | 17.2 | 20.5 | Regimen violation (frequency from QoD to QD) | ITI-alone, success, 4.4 mo |
| 2 | neg | NA | Intron 22 inversion | 1.6 | 1.6 | 0.2 | 24.6 | 24.3 | 14.1 | Regimen violation  (frequency of QD from ITI beginning) | ITI-alone, success,  4.6 mo |
| 3 | neg | NA | Large deletion/ duplication | 14.8 | 14.9 | 0.8 | 263.7 | 263.7 | NA | Withdrawal of consent | NA |
| 4 | neg | NA | Large deletion/ duplication | 3.4 | 9.5 | 73.8 | 37.8 | 37.8 | 256 | Lost Follow-up | ITI-alone, follow-up for 2 wk |
| 5 | neg | NA | Small insertion/deletion | 8.5 | 8.8 | 3.8 | 1.7 | 1.4 | 2.1 | Lost Follow-up | ITI-alone, follow-up for 2 mo |
| 6 | neg | NA | Unknown mutation | 13.7 | 13.7 | 0.2 | 1.2 | 0.9 | 0 | Regimen violation (FVIII 13 IU/kg. TiW) | ITI-alone,  success,  1.4 mo |
| 7 | neg | NA | Large deletion/ duplication | 2.3 | 4.3 | 24.6 | 25 | 25 | NA | Withdrawal of consent | NA |
| 8 | pos | 3.99 | Splice site mutation | 1.3 | 1.5 | 2.2 | 1.8 | 1.7 | NA | Withdrawal of consent | NA |
| 9 | neg | NA | Missense mutation | 4.6 | 5.5 | 11.0 | 3.4 | 3.4 | NA | Withdrawal of consent | NA |
| 10 | neg | NA | Intron 22 inversion | 0.9 | 1.8 | 11.7 | 70.4 | 40.8 | NA | Withdrawal of consent | NA |
| 11 | pos | 6.45 | Intron 22 inversion | 2.3 | 7.9 | 66.8 | 26.0 | 6.0 | NA | Withdrawal of consent | NA |
| 12 | neg | NA | Splice site mutation | 13.1 | 13.7 | 7.6 | 24.0 | 4.8 | NA | Withdrawal of consent | NA |
| 13 | neg | NA | Small insertion/deletion | 8.7 | 8.7 | 0.2 | 1.4 | 0.6 | NA | Withdrawal of consent | NA |

Abbreviations: ITI, immune tolerance induction; BU, Bethesda Unit; MMc, maternal microchimerism;

**Table 2. Sensitivity analysis of predictors of time to ITI success after exclusion of 32 patients with intron 22 inversion.**

| **Univariate analysis** | | ***p-*value** |
| --- | --- | --- |
| *F8* mutation (large deletion/ duplication and others) | | 0.002 |
| MMc (+/-) | | 0.336 |
| Historical peak inhibitor (<200BU/ml vs ≥200BU/ml) | | 0.016 |
| Age at ITI start (<5 years vs ≥5 years) | | 0.649 |
| Time interval from inhibitor detection to ITI (<12 months vs ≥12 months) | | 0.923 |
| Pre-ITI inhibitor (<10BU/ml vs ≥10BU/ml) | | <0.001 |
| Peak inhibitor during ITI (<200BU/ml vs ≥200BU/ml) | | 0.002 |
| **Multivariate analysis** | **HR (95% CI)** | ***p*-value** |
| MMc+ | 3.154 (1.197, 8.308) | 0.020 |
| Peak inhibitor during ITI <200BU/ml | 11.334 (2.494, 51.507) | 0.002 |
| Non-large deletion/duplication F8 mutations | 3.071 (1.244, 7.584) | 0.015 |
| Pre-ITI inhibitor <10BU/ml | 2.062 (1.069, 3.981) | 0.031 |

**Table 3. Sensitivity analysis of predictors of time to ITI success after exclusion of 14 patients with large deletion/duplication *F8* mutations.**

| **Univariate analysis** | | ***p-*value** |
| --- | --- | --- |
| MMc (+/-) | | 0.012 |
| Historical peak inhibitor (<200BU/ml vs ≥200BU/ml) | | 0.009 |
| Age at ITI start (<5 years vs ≥5 years) | | 0.828 |
| Time interval from inhibitor detection to ITI (<12 months vs ≥12 months) | | 0.686 |
| Pre-ITI inhibitor (<10BU/ml vs ≥10BU/ml) | | <0.001 |
| Peak inhibitor during ITI (<200BU/ml vs ≥200BU/ml) | | 0.007 |
| **Multivariate analysis** | **HR (95% CI)** | ***p*-value** |
| MMc+ | 2.812 (1.524, 5.187) | 0.001 |
| Peak inhibitor during ITI <200BU/ml | 5.038 (1.426, 17.800) | 0.012 |
| Pre-ITI inhibitor <10BU/ml | 2.504 (1.491, 4.205) | 0.001 |
